# Two new species of *Hirsutella* (Ophiocordycipitaceae, Sordariomycetes) that are parasitic on lepidopteran insects from China

**DOI:** 10.3897/mycokeys.82.66927

**Published:** 2021-08-09

**Authors:** Jiaojiao Qu, Xiao Zou, Wei Cao, Zhongshun Xu, Zongqi Liang

**Affiliations:** 1 College of Tea Sciences, Guizhou University, Guiyang, 550025, China Guizhou University Guiyang China; 2 Institute of Fungal Resources, College of Life Sciences, Guizhou University, Guiyang, 550025, China Guizhou University Guiyang China

**Keywords:** entomopathogenic fungi, *
Hirsutella
*, *
Ophiocordyceps
*, two new taxa

## Abstract

*Hirsutella* are globally distributed entomopathogenic fungi that offer important economic applications in biological control and biomedicine. *Hirsutella* was suppressed in favour of *Ophiocordyceps* affected by the ending of dual nomenclature for pleomorphic fungi in 2011. Currently, *Hirsutella* has been resurrected as a genus under Ophiocordycipitaceae. In this study, we introduce two new species of *Hirsutella*, based on morphological and phylogenetic analyses. *Hirsutellaflava* and *H.kuankuoshuiensis* are pathogenic on different species of larval Lepidoptera in China. *Hirsutellaflava* primarily differs from related species by its awl-shaped base; long and narrow neck, 24–40.8 × 2.2–2.5 μm; long and narrow cymbiform or fusoid conidia, 6.5–10 × 2.1–4.3 μm. *Hirsutellakuankuoshuiensis* has two types of phialides and distinctive 9.9–12.6 × 2.7–4.5 μm, clavate or botuliform conidia. The distinctions amongst the new species and phylogenetic relationships with other *Hirsutella* species are discussed.

## Introduction

The entomopathogenic fungal genus *Hirsutella* Pat. was erected by Patouillard (1892) based on the type species *H.entomophila*. The genus was introduced to the family Ophiocordycipitaceae and its sexual morph was linked to *Ophiocordyceps* (Sung et al. 2007a; Simmons et al. 2015). In *Hirsutella* sensu stricto, conidiation is synnematous and phialides typically have a swollen base that tapers abruptly into a long neck producing either a single conidium or 2–3 conidia coated with mucus. The colour of the synnemata ranges from ash-grey or brown to dark brown. The size and shape of the hyaline conidia vary from citriform to oblong, subcylindric, globose, rhombic, or reniform (Luangsa-ard et al. 2017; Quandt et al. 2014). These taxa are important pathogens of agricultural pests and are used as popular traditional medicine and a nutritious food in many Asian countries (Evans 1974; Quandt et al. 2014; Hyde et al. 2019). Several common species of *Hirsutella*, such as *H.thompsonii* and *H.rhossiliensis*, are potentially important biological control agents for nematodes and mites (Jaffee 1992; Van der Geest 2010; Hyde et al. 2019). Further uses involve the development and application of several effective bioactive secondary metabolites (Mazet and Vey 1995; Lang et al. 2005; Qu et al. 2017).

Research on *Hirsutella* originated in the 1920s. Through the 1950s, Speare (1920), Petch (1924) and subsequent researchers reported 25 new species of the genus. However, many of these species were not described in detail and lacked adequate drawings, as well as holotypes. In addition, many specimens were damaged or lost during wartime (Zou et al. 2016a). In the 1970s and 1980s, Miner, Samson and Evans re-examined the status of *Hirsutella* and established the modern scientific definition for the genus (Minter and Brady 1980; Evans and Samson 1982, 1984). Since the beginning of the 21^st^ century, the taxonomy, molecular evolution and phylogeny of *Hirsutella* have been addressed by a small number of Chinese and international studies, with sporadic reports of new species (Seifert 2004; Xiang et al. 2006; Zou et al. 2010). However, it is likely that further new species remain to be discovered, and specific information on insect hosts, pathogenicity and habitats are lacking (Sung et al. 2007a; Hoyos-Carvajal et al. 2009).

Quandt et al. (2014) proposed that *Hirsutella* should be suppressed in favour of *Ophiocordyceps* affected by the ending of dual nomenclature for pleomorphic fungi in 2011 (McNeill et al. 2012). *Ophiocordyceps* is the type genus in the family Ophiocordycipitaceae (Hypocreales, Sordariomycetes) (Sung et al. 2007a). The main characteristics of the sexual morphs of *Ophiocordyceps* are fibrous, hard, pliant-to-wiry, dark stromata with superficial to immersed perithecia (Sung et al. 2007a; Xiao et al. 2019). Most of the sexual species of *Ophiocordyceps* were transferred from the genus *Cordyceps* (Cordycipitaceae) by Sung et al. (2007a). Since many species of *Hirsutella* are closely related to *Cordyceps*, the asexual morphs in most of the species in *Ophiocordyceps* have hirsutella-like features (Kepler et al. 2013; Quandt et al. 2014; Maharachchikumbura et al. 2015, 2016). Therefore, *Hirsutella* was treated as a separate genus from *Ophiocordyceps* before the taxonomic revision (Sung et al. 2007a; McNeill et al. 2012; Quandt et al. 2014). For example, some new species only known from a *Hirsutella* morph have been accepted into *Ophiocordyceps* (Simmons et al. 2015a; Qu et al. 2018b).

In recent years, the taxonomic transitions of Ophiocordycipitaceae changed rapidly under the new rules. Quandt et al. (2014) included *Ophiocordyceps*, *Tolypocladium*, *Polycephalomyces*, *Purpureocillium*, *Drechmeria* and *Harposporium* in Ophiocordycipitaceae based on morphological and phylogenetic analyses. In the paper “Outline of Ascomycota: 2017”, the genus *Hymenostilbe* was added into the Ophiocordycipitaceae families (Wijayawardene et al. 2018). According to the latest taxonomic report, the number of genera included in Ophiocordycipitaceae has increased to ten, and among them, *Hirsutella*, *Paraisaria* and *Perennicordyceps* are new additions (Hyde et al. 2020). The taxonomic revision of Ascomycota is continuing. Further research into the phylogeny of these organisms is needed. Examples include investigating the new resources to supplement the available taxonomic information and perform phylogenetic research.

During an investigation of the genetic resources of entomopathogenic fungi in southwest China, we collected two specimens of Lepidoptera insects that were infected by fungi. Two hirsutella-like species were isolated and their gene sequences and morphological traits were shown to be related to *Hirsutella* sensu stricto. In this study, two new species of *Hirsutella* are introduced.

## Materials and methods

### Specimens

The specimens HKAS112884 and HKAS112885 were deposited at the Kunming Institute of Botany, Chinese Academy of Sciences (KIB), Kunming, China. The isolated strains of their asexual stage were deposited at the Institute of Fungal Resources of Guizhou University (**GZAC**), Guiyang, China. More information about these specimens is shown in Suppl. material 1: Table S1.

### Fungal isolation and culture

The fungi were isolated as described by Qu et al. (2018). The surface of specimens was rinsed with sterile water, followed by surface sterilisation with 75% ethanol for 3-5 s. Parts of the insect body were cut off and a piece of tissue was inoculated in haemocoel on a PDA plate for 20 days at 16 °C.

### LM and SEM observation

For light microscopy (LM) observations and imaging, the morphological characteristics of mycelia were observed using an optical microscope (OM, BK5000, OPTEC, Chicago, IL, USA) after staining with a lactic acid/phenol cotton blue solution. The captured images of new species were edited and digitally contrasted using Paint Shop Pro v. 5.0.1 (Corel, Ottawa, Canada).

Electron microscopy was performed as described by Qu et al. (2018). Briefly, 1 cubic cm of hyphae with conidia were cut from the fungus on PDA cultures, fixed with 4% glutaraldehyde at 4 °C overnight, and then washed three times with phosphate buffer saline (PBS) (137 mM NaCl, 2.7 mM KCl, 8.1 mM Na_2_HPO_4_ and 1.5 mM KH_2_PO_4_, pH 7.4) for 10 min each time. Fixed hyphae and conidia were dehydrated using 50%, 70%, 90% and 100% ethanol, 10 min for each concentration, and were finally dehydrated with super-critical carbon dioxide. After being sprayed with gold, the conidia and mucilage were examined by scanning electron microscopy (SEM) (S-3400N, Hitachi, Tokyo, Japan) and photographed.

### DNA extraction, PCR amplification and sequencing

Axenic and fresh mycelia (0.05–0.1 g) of the new species were transferred to 1.5 ml Eppendorf tubes for genomic DNA extraction using a Fungal DNA MiniKit (Omega Bio-Tek, Norcross, GA, USA). The universal known primers were used for PCR amplification: (1) NS1/NS4 for the partial small subunit ribosomal RNA gene region (SSU) (White et al. 1990), (2) LROR/LR5 for the partial large subunit rDNA gene region (LSU) (Vilgalys and Hester 1990; Rehner and Samuels 1994), (3) ITS4/ITS5 for the internal transcribed spacer gene region (ITS) (White et al. 1990), (4) 983F/2218R for the partial translation elongation factor 1-alpha gene region (TEF1α) (Sung et al. 2007b) and (5) CRPB1A/RPB1Cr for the partial RNA polymerase II largest subunit gene region (RPB1) (Castlebury et al. 2004).

### Molecular phylogeny

To construct a phylogeny of major lineages, 71 representative species were chosen to represent the ecological diversity of *Hirsutella* and *Ophiocordyceps* based on previous phylogenetic studies (Simmons et al. 2015b; Xiao et al. 2017; Qu et al. 2018; Xiao et al. 2019). *Tolypocladiuminflatum* and *T.ophioglossoides* were selected as the outgroup taxa and are classified within Ophiocordycipitaceae (Xiao et al. 2019). The sequences used in this study were combined with published data on hirsutella-like species and Ophiocordycipitaceae. All the other sequences were collected from GenBank and the accession numbers are shown in Table 1.

**Table 1.** GenBank accession numbers for sequences used in the phylogenetic analysis.

Species	Insecta	Voucher	GenBank accession no.				
			ITS	LSU	SSU	RPB1	TEF1α
Hirsutella cf. haptospora	Diptera: Itonididae	ARSEF 2228	KM652166	KM652118	KM652075	KM652041	KM652001
* H. changbeisanensis *	Homoptera: leafhopper	GZUIFR-hir160527	KY415578	KY415586			KY415592
* H. citriformis *	Hemiptera: Delphacidae	ARSEF 490	KM652151	KM652103			KM651987
*H.citriformis* (*Cixiidae*)	Hemiptera: Cixiidae	ARSEF 1035	KM652153	KM652105	KM652064	KM652030	KM651989
*H.citriformis* (*Psyliidae*)	Hemiptera: Psyllidae	ARSEF 2598	KM652155	KM652107			KM651991
* H. cryptosclerotium *	Hemiptera: Pseudococcidae	ARSEF 4517	KM652157	KM652109	KM652066	KM652032	KM651992
*** H. flava ***	Lepidoptera:	**GZUIFR-hir100627-1**	KY415598	KY415599		KY945366	KY415601
	Lepidoptera:	**GZUIFR-hir100627-2**	MF623036	MF623042			MF623046
	Lepidoptera:	**GZUIFR-hir100627-3**	MF623037	MF623043			MF623047
* H. fusiformis *	Coleoptera: Curculionidae	ARSEF 5474		KM652110	KM652067	KM652033	KM651993
* H. guyana *	Hemiptera: Cicadellidae	ARSEF 878	KM652158	KM652111	KM652068	KM652035	KM651994
* H. haptospora *	Acari: Uropodina	ARSEF 2226	KM652159			KM652036	KM651995
* H. illustris *	Hemiptera: Aphididae	ARSEF 5539	KM652160	KM652112	KM652069	KM652037	KM651996
* H. kirchneri *	Acari: Eriophyidae	ARSEF 5551	KM652161	KM652113	KM652070		KM651997
*** H. kuankuoshuiensis ***	Lepidoptera:	**GZUIFR 2012KKS3-1**	KY415575	KY415582		KY945360	KY415590
	Lepidoptera:	**GZUIFR 2012KKS3-2**	MF623038	MF623044			MF623048
	Lepidoptera:	**GZUIFR 2012KKS3-3**	MF623039	MF623045			MF623049
* H. leizhouensis *	Lepidoptera: Pyralidae	GZUIFR-hir140506	KY415573	KY415580			KY415587
* H. lecaniicola *	Hemiptera: Coccidae	ARSEF 8888	KM652162	KM652114	KM652071	KM652038	KM651998
* H. liboensis *	Lepidoptera: Cossidae	ARSEF 9603	KM652163	KM652115	KM652072		
* H. necatrix *	Acari	ARSEF 5549	KM652164	KM652116	KM652073	KM652039	KM651999
* H. nodulosa *	Lepidoptera: Pyralidae	ARSEF 5473	KM652165	KM652117	KM652074	KM652040	KM652000
* H. radiata *	Diptera	ARSEF 1369		KM652119	KM652076	KM652042	KM652002
H. repens *nom. inval.*	Hemiptera: Delphacidae	ARSEF 2348	KM652167	KM652120	KM652077		KM652003
*H.rhossiliensis* (*Heteroderide*)	Tylenchida: Heteroderidae	ARSEF 2931	KM652168	KM652121	KM652078	KM652043	KM652004
* H. rhossiliensis *	Tylenchida: Criconematidae	ARSEF 3747	KM652170	KM652123	KM652080	KM652045	KM652006
* H. satumaensis *	Lepidoptera: Pyralidae	ARSEF 996	KM652172	KM652125	KM652082	KM652047	KM652008
* H. sinensis *	Lepidoptera: Hepialidae	ARSEF 6282	KM652173	KM652126	KM652083	KM652048	KM652009
*H.strigosa* (*Cicadellidae*)	Hemiptera: Cicadellidae	ARSEF 2197	KM652175	KM652129	KM652085	KM652050	KM652012
*H.strigosa* (*Delphacidae*)	Hemiptera: Delphacidae	ARSEF 2044	KM652174	KM652128			KM652011
* H. subulata *	Lepidoptera: Microlepidoptea	ARSEF 2227	KM652176	KM652130	KM652086	KM652051	KM652013
*H.thompsonii* (*Eriophyidae*)	Acari: Eriophyidae	ARSEF 253	KM652179	KM652133	KM652088		KM652016
*H.thompsonii* (*Tetranychidae*)	Acari: Tenuipalpidae	ARSEF 3323	KM652188	KM652143	KM652096	KM652059	KM652024
H. thompsonii var. synnematosa	Acari: Tetranychidae	ARSEF 5412	KM652193	KM652148	KM652100		
H. thompsonii var. thompsonii	Acari: Eriophyidae	ARSEF 137	KM652177	KM652131	KM652087	KM652052	KM652014
* H. versicolor *	Hemiptera: Membracidae	ARSEF 1037		KM652150	KM652102	KM652063	KM652029
* Ophiocordyceps acicularis *	Coleoptera	OSC 110988		EF468804	EF468951	EF468853	EF468745
* O. agriotidis *	Coleoptera	ARSEF 5692	JN049819	DQ518754	DQ522540	DQ522368	DQ522322
* O. aphodii *	Coleoptera	ARSEF 5498		DQ518755	DQ522541		DQ522323
* O. appendiculata *	Coleoptera	NBRC 106960	JN943326	JN941413	JN941728	JN992462	AB968577
* O. brunneipunctata *	Coleoptera (Elateridae)	OSC 128576		DQ518756	DQ522542	DQ522369	DQ522324
* O. clavata *	Coleoptera	NBRC 106962	JN943328	JN941415	JN941726	JN992460	AB968587
* O. cochlidiicola *	Insect	HMAS199612	AB027377	KJ878884	KJ878917	KJ878998	KJ878965
* O. communis *	Coleoptera	NHJ 12581		EF468831	EF468973		EF468775
* O. dipterigena *	Diptera (adult fly)	OSC 151912		KJ878887	KJ878920	KJ879001	KJ878967
* O. elongata *	Lepidoptera (larva)	OSC 110989		EF468808		EF468856	EF468748
* O. entomorrhiza *	Lepidoptera	KEW 53484	JN049850	EF468809	EF468954	EF468857	EF468749
* O. evansii *	Hymenoptera (Pachycondylaharpax)	Ophsp 858		KC610770	KC610796	KP212916	KC610736
* O. forquignonii *	Diptera (adult fly)	OSC 151908		KJ878889	KJ878922	KJ879003	
* O. geometridicola *	Lepidoptera (Geometridae)	TBRC 8095		MF614648		MF614663	MF614632
* O. gracilis *	Lepidoptera (larva)	EFCC 8572	JN049851	EF468811	EF468956	EF468859	EF468751
* O. heteropoda *	Hemiptera (cicada nymph)	OSC 106404		AY489722	AY489690	AY489651	AY489617
* O. irangiensis *	Hymenoptera (adult ant)	OSC 128579		EF469076	EF469123	EF469089	EF469060
* O. konnoana *	Coleoptera (larva)	EFCC 7315			EF468959	EF468861	EF468753
* O. lanpingensis *	Hepialus (larva)	YHOS0707		KC417461	KC417459	KC417465	KC417463
* O. lloydii *	Hymenoptera (Camponotus)	OSC 151913		KJ878891	KJ878924	KJ879004	KJ878970
* O. macroacicularis *	lepidopterans (larvae)	NBRC 105888	AB968401	AB968417	AB968389		AB968575
* O. melolonthae *	Coleoptera (Scarabeidae larva)	OSC 110993		DQ518762	DQ522548	DQ522376	DQ522331
* O. multiperitheciata *	Lepidoptera (larva)	BCC 69008		MF614657			MF614641
* O. myrmicarum *	Formicidae (adult ant)	ARSEF 11864			KJ680150	KJ680151	JX566973
* O. nigrella *	Lepidoptera (larva)	EFCC 9247	JN049853	EF468818	EF468963	EF468866	EF468758
* O. pauciovoperitheciata *	Lepidoptera (larva)	TBRC 8106		MF614652			MF614633
* O. pseudoacicularis *	Lepidoptera (larva)	TBRC 8102		MF614646		MF614661	MF614630
* O. ramosissimum *	Lepidoptera (larva)	GZUHHN8	KJ028007		KJ028012	KJ028017	KJ028014
* O. robertsii *	Lepidoptera (Hepialidae larva)	KEW 27083		EF468826			EF468766
* O. sinensis *	Lepidopteran pupa	EFCC7287	JN049854		EF468971	EF468874	EF468767
* O. sporangifera *	Lepidoptera (Cossidae)	MFLUCC 18-0492	MH725818	MH725832	MH725814	MH727392	MH727390
* O. stylophora *	Coleoptera (Elateridae larva)	OSC 111000	JN049828	DQ518766	DQ522552	DQ522382	DQ522337
* O. xuefengensis *	Lepidoptera (Hepialidae larva)	GZUH2012HN11	KC631803		KC631788	KC631799	KC631794
* Tolypocladium inflatum *	Coleoptera (larva)	OSC 71235	JN049844	EF469077	EF469124	EF469090	EF469061
* T. ophioglossoides *	Fungi (Elaphomyces sp.)	NBRC 106332	JN943322	JN941409	JN941732	JN992466	

All the sequences were edited for multi-alignment using the BioEdit Sequence Alignment Editor v.7.0.5.3 (Hall 1999) with the Clustal X v.1.83 software package (Thompson et al. 1999). Gaps were excluded from the phylogenetic analysis based on previous research (Qu et al. 2018). The ITS, SSU, LSU, TEF1α and RPB1 regions were aligned in combined datasets using MAFFT v.7 (Katoh and Standley 2013, http://mafft.cbrc.jp/alignment/server/). The Akaike Information Criterion (AIC) in jModeltest 0.1.1 (Guindon and Gascuel 2003; Posada 2008) was used to select the nucleotide substitution model for each region. The combined data included a 4778 bp character set of the five regions and were analysed. Maximum likelihood phylogenetic analyses were conducted in RAxML (Stamatakis et al. 2008) with the recommended partition parameters to determine the best tree topology. The bootstrap support values were achieved after 500 search replicates and summarised in TreeGraph. Bayesian Posterior Probabilities (BPP) were estimated in MrBayes 3.1.2 (Ronquist and Huelsenbeck 2003) with the same partition parameters. In this analysis, two runs of four chains each were executed simultaneously for 5,000,000 generations, with sampling every 500 generations. TreeGraph was used to compute the BPP from a summary of 7,501 trees retained after a burn-in of the first 2,500 trees collected.

## Results

### Phylogenetic analyses

**Figure 1.**
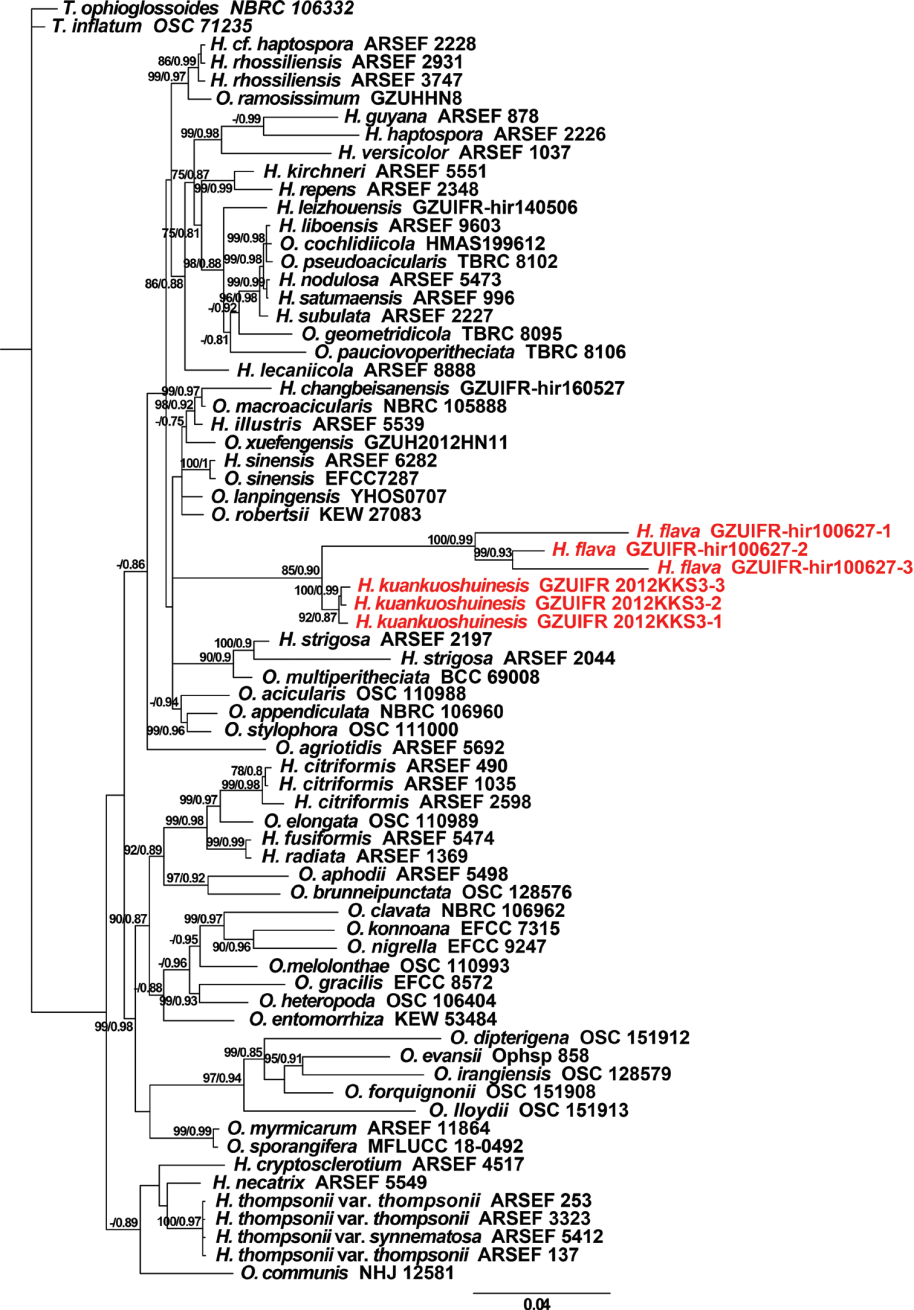
Phylogenetic tree of *Hirsutella* species combined with RPB1, *tef1*, ITS, 18S rDNA and 28S rDNA datasets, using the maximum likelihood method. Numbers below the branches are bootstrap percentage values, based on 10,000 replicates, ML/BPP.

The tree was constructed with maximum likelihood and Bayesian posterior probabilities with *Tolypocladiuminflatum* and *T.ophioglossoides* as the outgroup taxa based on RPB1, *tef1*, ITS, 18S rDNA and 28S rDNA gene datasets (SSU: 1391 bp, LSU: 903 bp, ITS: 721 bp, TEF1α: 946 bp and RPB2: 817 bp) (Fig. 1). In this phylogenetic tree, *Hirsutellaflava* and *H.kuankuoshuiensis* formed a separate clade from the other species with credible bootstrap values (85% ML and 0.90 PP), suggesting that these two species are truly related. Within a separate branch, *H.flava* and *H.kuankuoshuiensis* were allied with the *H.sinensis* and *H.strigosa* clade, distant from the other hirsutella-like species, particularly the *H.thompsonii* clade. A molecular phylogenetic analysis further confirmed the differences among the two new species and other related species. Based on the morphological characteristics and molecular phylogenetic analysis, these two new species are introduced as new members of *Hirsutella* species in the Ophiocordycipitaceae family.

### Taxonomy

#### Hirsutella flava

Taxon classificationFungiHypocrealesOphiocordycipitaceae

X. Zou, J.J. Qu, Z.A. Chen & Z.Q. Liang sp. nov.

D3899576-81FA-54BA-AAC9-AFE6F4964876

819552

Fig. 2

##### Diagnosis.

Characterised by phialides slender awl-shaped and tapered; a width of base 24–40.8 × 2.2–2.5 μm; tapering to narrow neck, 7.2–9 μm long × 0.5 μm wide. Conidia narrow cymbiform, long fusoid or limoniform, 6.5–10 × 2.1–4.3 μm.

##### Type.

China, Zhejiang Province, Tianmu Mountain National Nature Reserve (30°18'N, 119°28'E, approximately 600–1200 m a.s.l.), 27 June 2010, presented by Prof. Zhuan Chen. The holotype has been deposited at KIB (HKAS112884). Sequences from isolated strains (GZUIFR-hir100627-1, GZUIFR-hir100627-2 and GZUIFR-hir100627-3) have been deposited in GenBank.

##### Description.

**Figure 2.**
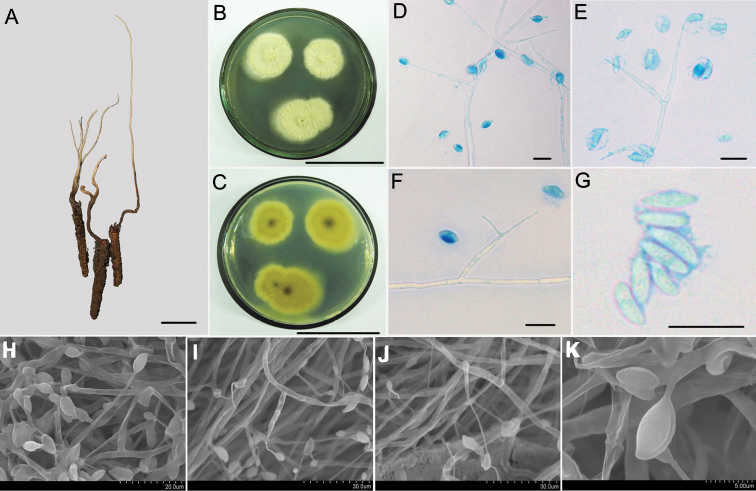
Morphological characteristics of *Hirsutellaflava***A** the infected insect specimens with a long and single synnemata (HKAS112884) **B, C** colonial morphology on PDA agar media for 20 d **B** shows the front of the colony and **C** shows the back of the colony **D–G**LM images of the general morphology of conidiogenous cells and conidia **H–K**SEM images showing conidiogenous cells and conidial structure; Scale bars: 1 cm (**A**); 5 cm (**B, C**), 10 μm (**D–G**); the rest of the bars are shown in the figure. LM, light microscopy; PDA, potato dextrose agar; SEM, scanning electron microscopy.

*Synnemata* extending from the head of insect, 3–10 cm × 0.5–1 mm, simple or irregularly branched, dark brown and changing to faint yellow toward the apex; no conidiation was observed (Fig. 2A). The fungus grows slowly at 22 ± 1 °C on Czapek-Dox agar medium to a diam. of 8–12 mm; the colony surface was flat and flocculent with white aerial hyphae. On PDA agar, fungal colonies grew quickly to a diam. of 15–23 mm after 20 d at 22 ± 1 °C, when the colonies were blanket-like with rough mycelia, radiating beam-like from the centre; centre lunate concave, pale yellow; colony surface with yellowish liquid exudation (Fig. 2B, C). *Mycelium* hyaline, smooth, septate, 3.6–4.5 μm wide. *Conidiogenous cells* form directly from the mycelial end, monophialidic or polyphialidic, and borne perpendicular or at acute angles (80°–85°) to the subtending hyphae. *Phialides* slender awl-shaped and tapered, width of the base 24–40.8 × 2.2–2.5 μm, tapering to a narrow neck, 7.2–9 μm long × 0.5 μm wide. *Conidia* narrow cymbiform, long fusoid or limoniform, 6.5–10 × 2.1–4.3 μm; single- or double-enveloped in a hyaline mucus, thickness 2.0–3.0 μm (Fig. 2D–K).

##### Host.

Larva of a species of Lepidoptera.

##### Habitat and distribution.

On decaying leaves in broadleaved forests, Zhejiang Province, China.

##### Etymology.

Refers to the yellow colour (Lat. ‘flava’) of the holotype and colony.

##### Teleomorph.

Unknown.

##### Remarks.

This species is allied with the *H.sinensis* and *H.strigosa* clade. The phialides of *H.flava* are subulate, and the necks are slenderer. In particular, the colony morphology of this fungus is unique among the *Hirsutella* species. The colony surface appears very rough, and the hyphae are gathered into outwardly radiating filamentous bundles of varying sizes.

#### Hirsutella kuankuoshuiensis

Taxon classificationFungiHypocrealesOphiocordycipitaceae

X. Zou, J.J. Qu & Z.Q. Liang sp. nov.

F0BE3307-2714-52AA-83B6-070FBE4087E0

819591

Fig. 3

##### Diagnosis.

*Hirsutellakuankuoshuiensis* differs from other species in this genus primarily by its clavate, narrow fusiform or botuliform conidia and subulate or slender columnar phialide.

##### Type.

China, Guizhou Province, Suiyang County, Kuankuoshui Nature Reserve (28°08'N, 107°02'E, approximately 1400 m a.s.l.), July 2012, collected by X. Zou. The holotype has been deposited at KIB (HKAS112885). Sequences from isolated strains (GZUIFR-2012KKS3-1, GZUIFR-2012KKS3-2 and GZUIFR-2012KKS3-3) have been deposited in GenBank.

##### Description.

**Figure 3.**
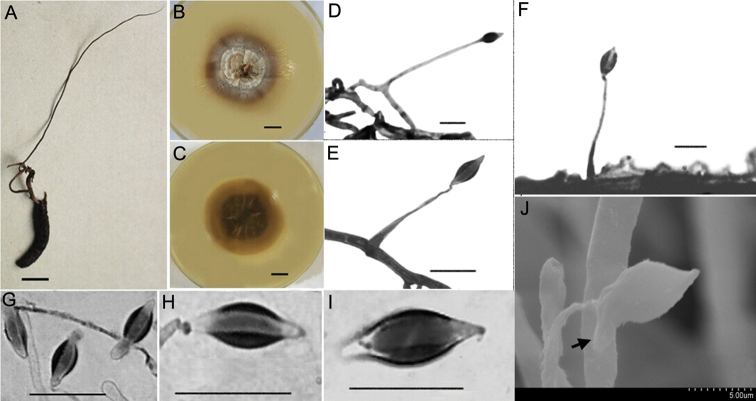
Morphological characteristics of *Hirsutellakuankuoshuiensis***A** the insect specimens with single and thin synnemata (HKAS112885) **B, C** colonial morphology on PDA agar media for 20 d **B** shows the front of the colony and **C** shows the back of the colony **D–G**LM images showing conidiogenous cells and conidia **D, E** the structure of conidiogenous cells on mycelia **F** the images of conidiogenous cells on synnemata (optical microscope) **H–J** conidial morphology (LM) **G** conidia with mucilage (SEM). Scale bars: 10 mm (**A–C**); bar of **G** was shown in the figure; the rest of the bars were 10 μm. LM, light microscopy; PDA, potato dextrose agar; SEM, scanning electron microscopy.

*Synnemata* are single, extending from the head of insect; 8.6 cm long, dark brown and changing to brown towards the apex; no conidiation was observed (Fig. 3A). The fungus spreads slowly on PDA agar at 20–22 °C and grows to a diam. of 22–30 mm after 14 d; the colony is round, centre of surface with brown dense bulges and grey-white sparse flocculent aerial hyphae. Colony margin is flat with radial groove; a large amount of brown pigment secreted into the medium causes the back of colony to appear dark brown; thickness 10–12 mm (Fig. 3B, C). *Mycelium* hyaline, smooth, septate, 1.5–3.0 μm wide. *Conidiogenous cells* monophialidic, hyaline, borne perpendicular or at an acute angle to the subtending hyphae. *Phialides* subulate or slender columnar, tapering gradually to a long and narrow neck, 30–45 × 1–3 μm long. *Conidia* clavate, narrow fusiform or botuliform without a diaphragm, 9.9–12.6 × 2.7–4.5 μm, single- or double-enveloped in a hyaline mucus, thickness 2.0–3.0 μm (Fig. 3D–J).

##### Etymology.

Referring to the locality of the specimen, kuankuoshui (Lat. ‘kuankuoshuiensis’).

##### Host.

Lepidoptera larva.

##### Habitat and distribution.

On the decaying leaves of broadleaved forests, Guizhou Province, China.

##### Teleomorph.

Unknown.

##### Remarks.

This species possesses two types of conidiogenous cells and long fusiform or clavate without diaphragm conidia (9.9–12.6 × 2.7–4.5 μm), which is extremely rare in *Hirsutella* species. In addition, *H.kuankuoshuiensis* could produce long thin synnemata on the culture media that contain few or no conidia.

## Discussion

Previous taxonomic studies have shown that the *Hirsutella* species are reconstructed in five main groups, and clustering taxa shared the same phialide structures (Simmons et al. 2015b; Qu et al. 2017; Qu et al. 2018). In general, the *H.nodulosa* lineage possesses phialides with apical helical twists. The *H.citriformis* clade is primarily represented by a squat ovoid base and a single slender neck. The *H.thompsonii* clade, the most widely studied hirsutella-like species and a potential biocontrol agent for mite pests, has a small cylindrical or round phialide, usually less than 25 μm, while the *H.sinensis* clade includes isolates that originate from a variety of taxa, including nematodes, mites and both hemi (Hemiptera) and holometabolous (Coleoptera, Lepidoptera) insect hosts (Simmons et al. 2015b). The majority of these species share a cylindrical base and an average phialide length greater than 40 μm. In our phylogenetic tree, these five typical branches of *Hirsutella* were more dispersed owing to the addition of more *Ophiocordyceps* species. *Hirsutellaflava* and *H.kuankuoshuiensis* formed a separate clade that is represented by the subulate phialides and narrow fusiform conidia and have a close relationship with the *H.sinensis* and *H.strigosa* clades. In addition, this separate clade is distant from the *H.thompsonii* and *H.citriformis* clades. Species in these clades primarily share similarly large phialides and long fusiform conidia (Qu et al. 2018).

The phylogenetic tree confirmed the distinction between two new species and extant species. Among the species with an awl-shaped base and a long narrow neck, *H.flava* differs in its subulate phialides (e.g. *H.danubiensis* Balazy et al., 2008; *H.tunicate*Ciancio et al., 2013), cylindrical phialides (e.g. *H.changbeisanensis* Liang, 1991; *H.strigosa* Petch, 1939) and two types of conidiogenous cells (e.g. *H.stilbelliformis* Evans & Samson, 1982; *H.shennongjiaensis*Zou et al., 2016b) (Suppl. material 1: Table S2). In addition, *H.flava* is unique in the colony morphology of isolated strains. The fungus spreads more quickly than other hirsutella-like species on PDA media, and the colony surface appears very rough, owing to the hyphae being gathered into outwardly radiating filamentous bundles of varying sizes. *H.flava* could be distinguished from similar species by the shape and size of the conidiogenous cells. Morphological comparisons of relevant taxa are shown in Suppl. material 1: Table S2.

*Hirsutellakuankuoshuiensis* possesses two types of conidiogenous cells and long fusiform or clavate conidia, which are unique to *Hirsutella*. Furthermore, this species can readily produce long thin synnemata on culture media, but it produces few or no conidia. There are five other species similar to this species: *H.shennongjiaensis* (Zou et al. 2016), H.stilbelliformisvar.stilbelliformis (Evans and Samson 1982), *H.sporodochialis* (Evans and Samson 1984), *H.subramanianii* (Samson and Evans 1985), and *H.zhangjiajiensis* (Liang et al. 2005). Among them, the conidia of *H.shennongjiaensis* are primarily rod-like and slender; H.stilbelliformisvar.stilbelliformis has a larger base with thorny phialides, greater than 50 μm long; *H.sporodochialis* has longer conidia; *H.subramanianii* has hymenopteran hosts and thinner stick-shaped conidia, 10–13.5 × 1.8–2.5 μm; and *H.zhangjiajiensis* conidia are lanceolate or resemble an orange segment (Suppl. material 1: Table S3). Within the framework of the available data for the genus, the phylogenetic tree and the morphological analysis confirmed the status of *Hirsutellaflava* and *H.kuankuoshuiensis* as new species.

## Supplementary Material

XML Treatment for Hirsutella flava

XML Treatment for Hirsutella kuankuoshuiensis

## Supplementary materials

Supplementary material 1 Tables S1–S3. A total of 71 taxa were selected to represent the morphological and ecological diversity of *Hirsutella* asexual morphs and *Ophiocordyceps* Data type phylogenetic data Explanation note The GenBank accession numbers of all species are shown in Table 1. https://binary.pensoft.net/file/575891This dataset is made available under the Open Database License (http://opendatacommons.org/licenses/odbl/1.0/). The Open Database License (ODbL) is a license agreement intended to allow users to freely share, modify, and use this Dataset while maintaining this same freedom for others, provided that the original source and author(s) are credited.Jiaojiao Qu, Xiao Zou, Wei Cao, Zhongshun Xu, Zongqi Liang
