# Supplementary material for: Two new species of Hirsutella (Ophiocordycipitaceae, Sordariomycetes) that are parasitic on lepidopteran insects from China
Source: MycoKeys. 2021 Aug 9;82:81–96. doi: 10.3897/mycokeys.82.66927 (PMC8367965; doi:10.3897/mycokeys.82.66927)
Supplement: Supplementary material 1 — Tables S1–S3. A total of 71 taxa were selected to represent the morphological and ecological diversity of Hirsutella asexual morphs and Ophiocordyceps [file mycokeys-82-081-s001.doc]

Table S1 Specimens information

| Specimen number | Collecting locations | Time | Specimen collector | Host | Asexual stage |
| --- | --- | --- | --- | --- | --- |
| HKAS112884 | Tianmu Mountain National Nature Reserve, Zhejiang Province, China (119°28' E; 30°18' N) | 27 June 2010 | Zhuan Chen | a species of Lepidoptera larvae | GZUIFR-hir100627-1 GZUIFR-hir100627-2 GZUIFR-hir100627-3 |
| HKAS112885 | Kuankuoshui Nature Reserve, Suiyang County, Guizhou Province, China (107° 02' E, 28° 08' N) | 12 July 2012 | Xiao Zou | a species of Lepidoptera larvae | GZUIFR-2012KKS3-1 GZUIFR-2012KKS3-2 GZUIFR-2012KKS3-3 |

Table S2 Morphological comparison among *Hirsutella flava* and its similar species

| Taxon | Phialides | Conidia | Host |
| --- | --- | --- | --- |
| ***Hirsutella flava* Zou** et al*.*  (this work) | Subulate phialides, 2.2−2.5×24−40.8 μm；  tapering to narrow ncek, 7.2−9×0.5 μm | Narrow cymbiform, long fusoid or limoniform, 6.5−10×2.1−4.3 μm | Cochlidiidae, Lepidoptera |
| *H. changbeisanensis* Liang (1991) | Phialidic, basally inflated, cylindric,  6.5−20×1.8−5.4 μm | Ellipsoid or orange segment, 3.6−5.5×3.0−4.5 μm | Cicadellidae, Homoptera |
| *Ophiocordyceps myrmicarum*  Simmons & Groden (2015) | Elliposoid base, 20.7−49.5 µm | Orange segment or narrow fusiform, 6.3−11.7×2.7−4.5 µm | Beetle,  Coleoptera |
| *H. strigosa* Petch (1939) | Subcylindrical, 36.0−50.0 μm | Cymbiform or orange segment, 8.0-12.0×3.0-5.0 µm | Cicadellidae, Homoptera |
| *H. eleutheratorum* Petch (1932) | Elliposoid base, 8−10×5−6 μm,  tapering into a long neck, 30−35 μm | Cymbiform to narrow ellipsoid, 4−7×1−2 μm | Coleoptera |
| *H. citriformis* Speara(1920) | Inflated bases, 6 −14×3−4 μm,  tapering into a single neck 20 μm | Cymbiform to fusoid, hyaline, 5.5 −8.5×1.5−2.5 μm | Cercopidae,  Homoptera |
| *H. danubiensis* Tkaczuk *et al*.(2008) | Phialides narrowly conical,  35–62.5 μm long, (average 48.4 μm), | Orange segment, 4.5–6.6×1.9–2.7 μm | Tetranychus urticae, Tetranychidae |
| *H. tunicata* Ciancio et al.(2013) | Phialidic, inflated awl-shaped base  10.0-23.0×2.5-5.0 μm | Thin, globular-apiculated envelope, 5.0−6.0×3.0−4.0 μm | Mite, Phytoseiidae |
| *H. sinensis* Liu et al.(1989) | Phialidic, tapering to narrow ncek,  17.3−47.6×3.2−5.4 μm | Long ellipsoidea, 5.4−14×3.2−4.3 μm | Hepialidac,  Lepidoptera |
| *H. exoleta* Petch (1936) | Inflated base, 20−40×5 μm, with a stout neck | Cymbiform, 9×2.5 μm, in mucous sheath | Lepidoptera (pupae) |
| *H. illustris* Minter & Brady (1980) | Plagiophialides, 50−100×7 μm,  with verruculose neck | Elliposoid, orange segment 15−20×4.5−6 μm | Lepidoptera (larva) |
| *H. gigantea* Petch (1937) | Oblong inflated base, 10−18×5−8 μm,  narrow neck, 10−20 μm | Elliposoid, 5−8×2−2.5 μm | Lepidoptera |
| *H. vandergeesti* Mietkiewski & Tkaczuk (2008) | Phialides narrowly conical, thickest at the base, 30.0−36.0×4−4.5 μm | Orange segment, 4.2-5.5×2.1-2.5 μm | Mite, Phytoseiidae |
| *O. robertsii* (Hook.) Berk (1855) | Awl-like, tapering to narrow ncek,  37−51×3.6−6.3 μm | Fusiform or cymbiform, with diaphragm, 9−14×4.5−5.5 μm | Lepidoptera (larva) |
| *H. darwinii* Evans & Samson (1982) | Slender awl shaped phialides;  12−45×1.5−2.5 μm | Narrowly fusiform, 4.5−11.5 × 1.5−2.0 μm | Araneida,  Arachnida |
| *H. nivea* Hywel-Jones(1997) | Monophialidic, subulate, 12.5−15.5×1−2 μm | Acerose, slender awl-shaped, 5.5−9.5× 0.5 μm | Cicadellidae,  Homoptera |
| *H. atewensis* Samson *et al*. (1982) | Polyphialidic, cylindric base, 9−15×2−4 μm | Fusiform, 8.0-12.0×4.5-5.0 μm | Cicadellidae,  Homoptera |
| *H. besseyi* Fisher(1950) | Acuminate, 2.5−4.9×21.6−66.4 μm | Narrowly elongate to citriform, 2.5−4.9×21.6−66.4 μm | Diaspidae,  Homoptera |
| *O. xuefengensis* Wen *et al.*(2013) | A & B; Cylindrical & awl-like,  24.3−41.4×2.7−3.6 µm | Orange segment or elliposoid，5.4−8.1×2.25−3.6 µm | Hepialidac,  Lepidoptera |
| *H. shennongjiaensis* Liang & Liu (2015) | A & B; Cylindrical & awl-like, 13−29×0.5−1 μm; 16−29×1−2.5 μm; | Sausage-shaped，6−12×4−6 μm | Earwig,  Dermaptera |
| *O. macroacicularis* Zhou *et al*.(2015) | A & B; Cylindrical & awl-like, 1.8−6.3 μm or 21.0−63.0 μm | Orange segment or oval,8.1−10.8×2.7−5.4 μm | Lepidoptera (larva) |
| *H. stilbelliformis* var*. stilbelliformis* Evans & Samson (1982) | A & B; Base inflated, ellipsoid & awl-like, 10.0−16.0 μm or 75.0−150.0 μm | Clavate, 7.0−9.0×1.5−2.2; Ovoid, 8.0−12.0×4.5−5.0 μm | Formicinae, Hymenoptera |
| *H. yunnanensis* var. *yunnanensis*  Z.Q. Liang & A.Y. Liu | globular base, 14.5 × 3−5.4 μm  tapering into a long neck | fusiform, clavate or orange-shaped, 4.8−6 × 1.5−2 μm | *Deileptenia* sp. |
| *H. polycolluta* Z. Q. Liang (1991) | Cylindrical base, 5−10 × 0.5−1.5 μm, multi-neck | fusiform, orange-shaped, 5−7.5(−8.5) × (1.25−)1.5−2 μm | Lepidoptera (larva) |
| *H. subulata* Petch (1932) | Phialides, base 4−8 × 3−5 μm, slender neck | Narrow ellipsiod, 4−8 × 1.5−2.5 μm | Lepidoptera (larva) |
| *H. hunanensis* Z.Q. Liang (2005) | Cylindrical or pear-shaped base, 5−10 × 0.5−1.5 μm | Long ellipsoid, 6×1.5−2 µm | Lepidoptera (larva) |

Table S3 Morphological comparison among *Hirsutella kuankuoshuiensis* and its similar species

| Species | Phialides | Conidia | Host |
| --- | --- | --- | --- |
| *Hirsutella necatrix* Miner et al.  (1983) | A & B; Base inflated & awl-like,  5.5*−*9.0 μm or 11.0*−*19.0 μm | Ovoid or ellipsoid,  3.0*−*4.0×2.5*−*3.0 μm | Acari, Eriophyidae |
| *H. shennongjiaensis* Zou et al.  (2016) | A & B; Cylindrical & awl-like | Sausage-shaped,  6.3*−*10.8×3.6*−*6.3 μm | Earwig, Dermaptera |
| *H. zhangjiajiensis* Liang & Liu  (2005) | A & B; Base inflated, ellipsoid &  awl-like, 30.0*−*52.0 μm | Lanceolate or orange  segment, 4.5*−*10.0×1.0*−*2.5 μm | Pupa, Lepidoptera |
| *H. strigosa* Petch (1939) | B; Subcylindrical, 36.0*−*50.0 μm | Cymbiform or orange  segment, 8.0*−*12.0×3.0*−*5.0 μm | Cicadellidae,  Homoptera |
| *Ophiocordyceps macroacicularis*  Zhou et al.(2015) | A & B; Cylindrical & awl-like,  1.8*−*6.3 μm or 21.0*−*63.0 μm | Orange segment or oval,  8.1*−*10.8×2.7*−*5.4 μm | Larva, Lepidoptera |
| *Oph. myrmicarum*  Simmons & Groden (2015) | B; awl-like, 20.7*−*49.5 µm | Orange segment or narrow fusiform, 6.3*−*11.7×2.7*−*4.5 µm | Beetle, Coleoptera |
| *H. sporodochialis*  Evans & Samson (1984) | A & B; Base inflated, flask-shape &  awl-like, 50.0*−*80.0 μm | Fusiform, 10.0*−*27.0×3.5*−*4.0 μm | Formicinae, Hymenoptera |
| *H. eleutheratorum* Petch (1932) | A; elliposoid, base 8*−*10×5*−*6 μm,  tapering into a long neck, 30*−*35 μm | cymbiform to narrow ellipsoid,  4*−*7×1*−*2 μm | Coleoptera |
| *H. stilbelliformis* var*. stilbelliformis*  Evans & Samson (1982) | A & B; Base inflated, ellipsoid & awl-like,  10.0*−*16.0 μm or 75.0*−*150.0 μm | Clavate, 7.0*−*9.0×1.5*−*2.2 μm;  Ovoid, 8.0*−*12.0×4.5*−*5.0 μm | Formicinae, Hymenoptera |
| *H. subramanianii* Samson & Evans (1985) | A & B; Base inflated, ellipsoid & subulate, 15*−*25×2.5*−*3.5 μm | obclavate to curved,  10.0*−*13.5×1.8*−*2.5 μm | Formicidae  Hymenoptera |
| ***H. kuankuoshuiensis*** Zou et al.  (this work) | A & B; slender columnar or short base inflated, 30*−*45×1*−*3 μm or 23*−*27 μm | narrow fusiform or botuliform,  9.9*−*12.6×2.7*−*4.5 μm | Larva, Lepidoptera |
